# Feasibility and Preliminary Effects of Ballet-Based Group Dance Intervention in Relapsing–Remitting Multiple Sclerosis: A Pilot Study

**DOI:** 10.3390/jcm14238612

**Published:** 2025-12-04

**Authors:** Daniela Ivaldi, Roberta Lombardo, Gabriele Triolo, Giovanni Restuccia, Carla Susinna, Lilla Bonanno, Carmela Rifici, Giangaetano D’Aleo, Edoardo Sessa, Angelo Quartarone, Viviana Lo Buono

**Affiliations:** IRCCS Centro Neurolesi Bonino-Pulejo, SS113 Via Palermo C. da Casazza, 98124 Messina, Italy; daniela.ivaldi@irccsme.it (D.I.); gabriele.triolo@irccsme.it (G.T.); giovanni.restuccia@irccsme.it (G.R.); carla.susinna@irccsme.it (C.S.); lilla.bonanno@irccsme.it (L.B.); carmela.rifici@irccsme.it (C.R.); giangaetano.daleo@irccsme.it (G.D.); edoardo.sessa@irccsme.it (E.S.); angelo.quartarone@irccsme.it (A.Q.); viviana.lobuono@irccsme.it (V.L.B.)

**Keywords:** ballet, group ballet-based intervention, motor rehabilitation, cognition, RRMS, feasibility, well-being

## Abstract

**Background:** Group-based dance interventions (GBDIs) have emerged as a promising approach to rehabilitation for neurological disorders. This pilot study evaluated the feasibility and preliminary effects of a GBDI on motor function, cognition, fatigue, and quality of life in individuals with Relapsing–Remitting Multiple Sclerosis (RRMS). **Methods:** The intervention consisted of two 60-min ballet sessions per week over 10 weeks, structured as 10 min of warm-up, 40 min of ballet exercises, and 10 min of stretching. Assessments were conducted at baseline (T0) and post-intervention (T1). Concerning motor measures, balance was assessed using the Mini-BESTest; gait performance was evaluated through the 6-min walk test (6MWT), four square step test (FSST), and figure-of-8 walk test (F8WT); upper limb motor functions were assessed using the box and block test (BBT) and 9-hole peg test (9HPT). Regarding cognitive functions, the Rey auditory verbal learning test (RAVLT), symbol digit modalities test (SDMT), and trail making test A and B (TMT-A/B) were administered, while fatigue and quality of life were assessed using the modified fatigue impact scale (MFIS) and the Short Form survey-36 (SF-36), respectively. **Results:** At T1, participants improved in Mini-BESTest (+17.5%), 6MWT (+7.3%), and BBT dominant hand (+6.9%). Performance also improved on the following cognitive tests: RAVLT Immediate Recall (+5.9%), RAVLT Delayed Recall (+20.3%), SDMT (+47.4%), TMT-A (−21.2%), and (TMT-B −24.5%). **Conclusions:** The very small sample size (*n* = 4) and the lack of a control group probably restrict the generalizability of the findings. Consequently, the results obtained by this pilot study should be considered exploratory and hypothesis-generating rather than definitive evidence of a robust benefit. Future studies should confirm these findings by enlarging the intervention cohorts and adopting a randomized controlled design. In this sense, a 10-week GBDI may provide a solid base for a safe and promising dance-based rehabilitation program that could lead to improvements in motor, cognition, and psychosocial spheres in people with RRMS.

## 1. Introduction

Multiple sclerosis (MS) is an inflammatory and neurodegenerative disease of the central nervous system. Over time, it leads to variably progressive disability through the involvement of the motor, sensory, and cerebellar pathways. Globally, 2.8 million people were affected by MS in 2020 [1], with a typical onset in early adulthood and a substantial impact on activities of daily living and social participation. The clinical presentation is heterogeneous. Common motor symptoms include walking impairment, muscle weakness, balance deficits, spasticity, fatigue, and reduced cardiorespiratory fitness, which contribute to risk of falls and participation restrictions [2,3]. Regarding the cognitive domain, people with MS (pwMS) encompass slowed information processing speed and deficits in attention/executive function and memory; those symptoms are strongly linked to poorer quality of life and difficulties with employment/participation [4].

Based on clinical course, it is possible to divide MS into clinically isolated syndrome, relapsing–remitting (RRMS), primary progressive, secondary progressive, and radiologically isolated syndrome [5,6]. RRMS is the most common form of MS [7], characterized by no continuous progression between relapses leading to a less severe clinical manifestation and a more preserved quality of life in many cases. Over the past two decades, structured exercise and physiotherapy-based rehabilitation have become core non-pharmacological components of comprehensive MS care. Evidence suggests that physical exercise can improve balance, walking ability, and endurance, reduce fatigue, and enhance health-related quality of life in pwMS. Accordingly, clinical guidelines recommend regular aerobic and resistance training tailored to disability level and delivered with appropriate progression and monitoring [8,9]. Within these therapeutic frameworks, dance-based rehabilitation has gained attention as an innovative approach for managing neurological disorders. Dance is a multimodal art form that provides an effective therapy for addressing gait and balance impairments in older adults as it combines multi-sensory elements, including proprioceptive, visual, auditory, and motor control techniques [10], and functional mobility [11,12]. Gait improvements have been reported across a broad range of dance styles, showing improvements in stride velocity [13], walking endurance [14,15], and muscle function [16]. Balance improvements are reported in a similarly wide variety of dance styles including contemporary [17,18], jazz [19], ballroom [16], salsa [13], tango [20,21,22,23], and traditional folk dance [24,25,26]. Dance integrates rhythmic movement, motor coordination, postural control, and cognitive engagement, offering a multimodal stimulus that extends beyond traditional physiotherapy [27].

Consequently, dance can be conceptualized as a multimodal motor learning paradigm that engages wide cortical and subcortical networks supporting complex motor mechanisms and higher-order cognition [28]. Evidence from neuroimaging studies suggests that performing spatially patterned, bipedal, rhythmic movements results in the consistent activation of a large-scale network, including primary motor and premotor cortices, supplementary motor area (SMA), parietal regions, cerebellum, basal ganglia, and limbic structures [28,29,30]. Long-term dance training is also associated with functional and structural changes within cortico-basal ganglia loops and sensorimotor networks, which could be interpreted as experience-dependent plasticity supporting the organization and integration of complex whole-body movements [31,32]. In addition, when movements are performed in synchrony with musical rhythm, as in dance, there is a strong engagement of the “sensorimotor synchronization” circuitry composed of cerebellum, basal ganglia, and motor cortices, which is critical for both rhythmic timing and predictive control of action [30,33].

Previous studies in neurological populations have highlighted that integrating social and musical components into group-based dance programs can amplify both motor and psychosocial outcomes, fostering long-term adherence and promoting a more multifactorial rehabilitative experience [27]. Evidence from Parkinson’s disease and stroke indicates that dance can improve balance, gait, mobility, and quality of life by combining motor training with musical and social components [27,34]. Among different dance forms, ballet may offer unique rehabilitative opportunities for pwMS. Ballet exercises emphasize postural alignment, balance, and controlled movements of both the upper and lower limbs, often combined with rhythmic sequencing and spatial orientation [35]. These features directly target functional domains frequently impaired in MS, such as stability, gait control, and coordination. In addition, the artistic and expressive dimensions of dance may enhance cognition, motivation, adherence, and psychosocial well-being [36]. The group-based nature of dance further provides opportunities for social interaction and peer support, which are particularly relevant for pwMS who often report reduced participation and social isolation [27,33]. In this sense, a dance-based intervention combines physical activity, cognitive challenge, and social interaction; hence, it could be considered a form of environmental enrichment [37,38]. Experimental and translational work indicates that enriched environments increase the expression of neurotrophic factors such as brain-derived neurotrophic factor (BDNF), promote hippocampal neurogenesis and synaptic plasticity, and exert neuroprotective effects against cognitive decline [31,39]. Considering the complex motor and cognitive impairments experienced by pwMS and the potential of dance-based interventions to address these domains, ballet may represent a structured and motivating group-based rehabilitation approach [36]. Nevertheless, evidence regarding the feasibility and effects of ballet in pwMS remains limited [35].

In this context, we conducted a pilot feasibility study of a group-based dance intervention (GBDI) for pwMS with RRMS to determine whether a GBDI can be delivered safely and acceptably in a rehabilitation setting and to explore preliminary motor and cognitive changes to inform future controlled trials. Here, we define feasibility primarily as intervention acceptability, the extent to which the program is well-tolerated, experienced as pleasant and engaging, and easy to integrate into patients’ routines compared with traditional physiotherapy, together with retention, adherence, safety (adverse events), standardized satisfaction measures, and participant feedback.

## 2. Materials and Methods

Six adult participants with an established RRMS diagnosis [40] were recruited from the MS clinic of IRCCS Neurolesi Bonino-Pulejo (Messina, Italy) during routine outpatient visits from December 2024 to April 2025. The intervention started in May 2025 and ended in July 2025. Thus, the entire study duration was 8 months. Eligible participants were between 18 and 65 years and had an Expanded Disability Status Score (EDSS) ≤ 4.5. Other eligible criteria included comparable age and residence in the same city. Exclusion criteria included a history of other primary neurologic or psychiatric disorders, severe cognitive impairment, and concurrent participation in other structured rehabilitation programs. Participants with other comorbidities that could interfere with safe participation during the intervention, such as the inability to walk without aids, were also excluded. None of the participants had previous experience with dance lessons. Two participants dropped out of the study: one shortly after the first session and one close to the end of the intervention. Thus, four out of six participants completed the intervention. Participants provided written informed consent to participate in the study. Written informed consent was also obtained from the individuals for the publication of any potentially identifiable images or data included in this article. The study was approved by the Ethics Committee of IRCCS Centro Neurolesi Bonino Pulejo (n. E40/23, 07/02/23).

### 2.1. Intervention

A GBDI was conducted by a physiotherapist with established experience in ballet teaching. The intervention consisted of a dance-based training protocol specifically designed for pwMS. Dance sessions were held two times weekly for 10 weeks; each one lasted approximately 60 min. The sessions were engaging and enjoyable, encouraging participants to explore movements and express themselves through ballet. The training combined rhythmic movement, postural control, balance tasks, and gait activities, progressively adapted to participants’ abilities. Each session was structured as follows: 10 min of warm-up exercises, 40 min of ballet steps and choreography, and 10 min of stretching, as illustrated in Table 1.

The difficulty of the dance steps increased progressively throughout the program, and part of the time dedicated to dance was spent on executing the choreography. Different steps were introduced in each session, and the complete choreography was repeated during the final 4 sessions. Furthermore, after 10 weeks, ballet steps were executed exclusively in the standing position. Classical music was selected to accompany the practice of dance steps, whereas a modern jazz dance piece was chosen for the choreography. Relaxing music was used during warm-up and stretching. During this phase, exercises included the use of elastic bands and incorporated both open and closed kinetic chain movements, with a chair support when necessary.

### 2.2. Outcome Measures

A comprehensive clinical assessment, including motor and neuropsychological tests, was performed at baseline (T0) and at 10 weeks (T1) by blinded trained clinicians. Motor outcomes were explored across multiple domains. Balance was assessed with the Mini-BESTest, while dynamic balance and functional mobility were examined using the four square step test (FSST) and the figure-of-8 walk test (F8WT). Walking endurance was evaluated with the 6-min walk test (6MWT). Upper limb dexterity was explored using the box and block test (BBT) and the 9-hole peg test (9HPT). Neuropsychological assessment included the Rey auditory verbal learning test (RAVLT) to evaluate verbal episodic memory, which includes immediate recall (IR) and delayed recall (DR) measures as indices of encoding and retrieval; the symbol digit modalities test (SDMT) to assess information processing speed; and the trail making test A (TMT-A) and B (TMT-B) to measure attention, processing speed, and executive functioning. Raw scores of the neuropsychological tests were successively adjusted according to the available Italian normative data. In addition, fatigue was measured with the modified fatigue impact scale (MFIS), and health-related quality of life was assessed through the 36-Item Short Form Health Survey (SF-36).

### 2.3. Adherence and Adverse Events

Adherence to the intervention was recorded by the trainer through a digital file recording presence at each session. Adverse events (e.g., falls) were self-reported and recorded by the dance instructor.

### 2.4. Participants Satisfaction

On the final session, overall satisfaction with the intervention was measured using the 8-item Client Satisfaction Questionnaire (CSQ-8) and a semi-structured interview was administered. The CSQ-8 comprises 8 items, each rated on a 4-point scale (1–4), with higher scores indicating greater satisfaction [41]. The participants were also interviewed using predetermined open-ended questions regarding exercises and class setting to explore their experience with the GBDI. An audio recording was conducted, and subsequently, the responses were transcribed from the recording.

### 2.5. Statistical Analysis

This pilot study adopted a feasibility-first analytic strategy. Descriptive distributions at T0 and T1 are reported as medians and interquartile ranges (IQRs), and clinical outcomes were treated as exploratory. For each outcome, pre- and post-intervention values were obtained for participants who completed the protocol, and within-participant change was quantified as the paired Hodges–Lehmann (HL) estimate of T1-T0 together with its 95% confidence interval. The HL estimator was selected because it provides a robust estimate of central paired change in very small samples without assuming normality. We did not perform null-hypothesis significance testing or report *p*-values, in line with the feasibility-focused and hypothesis-generating nature of this pilot study. Because different measures improve in opposite numerical directions, all outcomes were sign-aligned before plotting so that positive values consistently represent change in the clinically favorable direction. Specifically, for outcomes in which lower values indicate improvement (gait and mobility times, upper limb dexterity times, fatigue scores), paired differences were multiplied by -1 so that improvement corresponded to a positive HL estimate. For outcomes in which higher values indicate improvement (balance, processing speed, perceived quality of life), the raw paired difference T1-T0 was used. This harmonization allowed direct visual comparison across heterogeneous clinical domains. All analyses were performed in R (version 4.4.2).

## 3. Results

Six individuals with pwMS were enrolled; two discontinued before T1, yielding four completers available for analysis (retention 66.7%). One participant withdrew after the first session because of scheduling incompatibility, and another discontinued near the end of the program, after the sixteenth session, due to an intercurrent surgical procedure. Baseline clinical and demographic characteristics are summarized in Table 2. Among the four completers, age ranged from 31 and 45 (M 39.25; SD 6.55), sex ratio was 1:1, EDSS ranged from 1.5 and 4.5 (M 4; SD 0.5), and disease duration comprised between 1 and 10 years (M 8.75; SD 1.5). Mean attendance across completers was 80.8% of the scheduled sessions. No adverse events were reported during the intervention. Any supplementary information regarding individual demographic and clinical characteristics can be found in Appendix A.

Feasibility and acceptability outcomes supported the tolerability and perceived value of the intervention. Satisfaction with the program, as measured by CSQ-8, is summarized in Table 3. All participants rated the overall quality of the intervention as at least “moderately good”, and 50% rated it “excellent”. All participants reported that the service met their needs at least “moderately” and all indicated that they received the kind of service they wanted. Willingness to recommend the intervention to others and willingness to return for similar services in the future were also universally endorsed at a “moderate” or “excellent” level, and most participants reported that the intervention helped them deal more effectively with their problems.

Exploratory clinical outcomes suggested domain-specific changes from baseline (T0) to post-intervention (T1). Motor performance, including balance, gait, and mobility, is reported in Table 4. Participants showed higher Mini-BESTest scores at T1 compared with T0, shorter completion times on dynamic balance and mobility tasks such as the FSST, and increased walking endurance on the 6MWT. Performance on the F8WT remained essentially stable. Upper limb function showed a more heterogeneous pattern, BBT scores increased for the dominant hand and were stable for the non-dominant hand, whereas 9HPT times showed side-specific variability.

Neuropsychological outcomes, fatigue, and health-related quality of life are summarized in Table 5. Participants showed higher performance in verbal learning and delayed recall on the RAVLT, improved information processing speed on the SDMT, and faster completion times on attention and executive functioning tasks (trail making test A and B). Self-reported fatigue decreased on the MFIS. In addition, perceived vitality and mental health improved on the SF-36, whereas general health perception remained stable.

**Figure 1:**
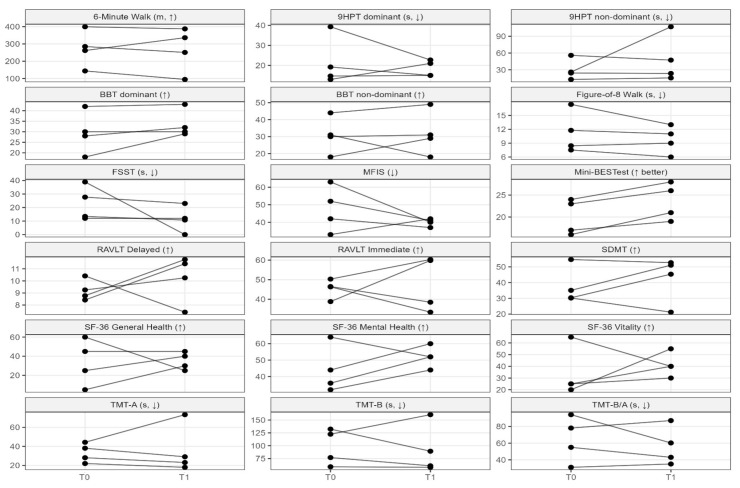
Individual pre–post trajectories across motor, cognitive, fatigue, and quality-of-life outcomes. Plot of participant-level trajectories from baseline (T0) to post-intervention (T1). Each line connects paired measurements from the same participant. Values are shown on the original clinical scales.

Individual participant trajectories from T0 to T1 are shown in Figure 1. Each panel reports paired pre–post values for a specific clinical domain, allowing for a visual inspection of both the direction and the consistency of change across participants.

**Figure 2:**
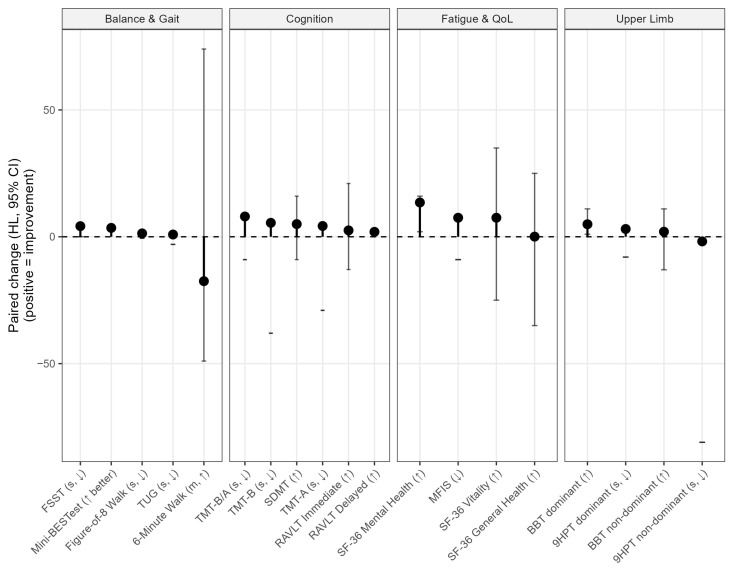
Domain-specific paired change from baseline to post-intervention. For each outcome, the paired Hodges–Lehmann (HL) estimate of change from T0 to T1 is also reported. Each point represents the HL estimate of change for that specific outcome, vertical bars represent the 95% confidence interval, and the horizontal dashed line at zero denotes no change. Outcomes are grouped by functional domain (balance and gait, cognition, fatigue and quality of life, and upper limb).

To provide an integrated view across domains and to account for the fact that some measures improve when scores increase while others improve when times decrease, within-participant paired change from T0 to T1 was also summarized using the HL estimator with 95% confidence intervals. These values are shown in Figure 2. For each outcome, the paired difference T1–T0 was computed, and for measures in which lower values indicate better performance (mobility tests, manual dexterity times, fatigue scores), the sign of the difference was inverted so that all improvements are plotted as positive values. Figure 2 shows the outcomes by functional domain.

### Semi-Structured Interview

Qualitative feedback obtained through a semi-structured interview was consistent with these questionnaire data. Participants described intense bodily engagement and temporary relief from illness-related self-awareness, “I felt like I was working my whole body and I forgot about my condition”; a sense of psychological safety and identification with peers, “For the first time in clinic I felt understood and not judged”; and anticipatory motivation, “Thinking about going to class made me feel more light-hearted”. Together with the absence of adverse events and the observed adherence, these findings indicate that the intervention was deliverable, acceptable, and well-tolerated in this cohort of pwMS and suggest perceived benefits that extend beyond motor function alone.

## 4. Discussion

The present study explored the feasibility and therapeutic efficacy of GBDIs in pwMS. Our results show high feasibility and acceptability in a 10-week structured program; this is supported by strong adherence, acceptable retention, absence of adverse events, and positive satisfaction ratings. These findings led to improvements across multiple motor domains, including balance, gait performance, and upper limb function. In addition, the participants showed improvement in cognitive abilities, fatigue, and perceived quality of life. These findings are in line with previous studies reporting that ballet training not only provides motor benefits but also enhances mental, cognitive, and social well-being [42]. Ballet involves specific movements that require precise spatial and temporal coordination of multi-joint limb movements combined with postural control [43], which may help explain the observed functional gains. Improvements in Mini BESTest score and FSST time were particularly noteworthy, given the high prevalence of postural instability in pwMS and its well-established association with falls and reduced mobility. Balance impairment is indeed recognized as one of the most debilitating symptoms for pwMS [44,45]. Gait performance also improved, with participants covering greater distances in the 6MWT, while F8WT time remained stable, suggesting that improvements were recognizable during longer walking-based assessments. Ballet trains postural stability through a mix of static and dynamic processes that may translate to improved gait and mobility [46]. Globally, these outcomes may reflect enhanced motor coordination, endurance, and dynamic stability, fostered by repeated exposure to rhythmic walking and direction changes integrated into the dance protocol. Upper limb function showed mixed results. Manual dexterity, measured by the 9HPT, did not show a clear improvement: median completion times increased for both hands, with larger changes in the non-dominant hand. Conversely, increased performance at BBT demonstrated moderate gains in the dominant hand. This asymmetry may be explained by differences in task demands, the specificity of motor improvements, or compensatory strategies adopted by participants. Further improvements were observed in the cognitive domains assessed. The increase in SDMT, TMT-A, and TMT-B scores may indicate an improvement in information processing speed and executive functioning. Each ballet session requires participants to encode, retain, and update sequences of movements, including the order of steps, their timing with the music, and their spatial orientation in the room. Therefore, this continuous sequence learning engages working memory, selective and divided attention, cognitive flexibility, and error monitoring [27,29]. Neurocognitive studies indicate that dance and complex motor sequence learning recruit fronto-parietal executive networks, premotor and SMA regions, and medial temporal structures implicated in sequence and episodic learning [27,47]. Regarding RAVLT, the present protocol did not involve the direct practice of word-list learning. Thus, gains in the RAVLT score may reflect domain-general hippocampal-dependent plasticity rather than a task-specific training effect. Ballet sessions involved sustained whole-body movement at light-to-moderate aerobic intensity. Aerobic exercise in pwMS has been shown to increase hippocampal volume, enhance hippocampal functional connectivity, and improve memory performance on list-learning tasks comparable to the RAVLT, as demonstrated in pilot and subsequent targeted trials of cycling or walking interventions [48,49,50]. Within this framework, repeated practice of spatial–temporal movement sequences during ballet may strengthen medial temporal–frontal networks that support episodic and verbal learning, while the aerobic and enriched nature of the sessions facilitates a more favorable neurotrophic and inflammatory milieu for hippocampal plasticity. These effects are likely mediated by the multimodal nature of dance, which requires participants to integrate sensory input, coordinate motor output, and maintain rhythmic synchronization with music, performing complex movement patterns while fulfilling esthetic goals. Improvements in cognitive functioning may reflect the potential of dance, particularly ballet, to train attention during motor tasks, thereby reducing cognitive load and allowing for more efficient motor performance [51].

In line with motor and cognitive gains, reductions in fatigue and improvements in self-reported health-related quality of life were observed. Notably, vitality and mental health domains showed the largest improvements, highlighting the potential psychosocial value of incorporating music and artistic expression into rehabilitation. This is consistent with previous research showing that engaging, enjoyable interventions are more likely to sustain participation and promote adherence [52]. Regarding the overall “quality assessment” of this intervention, several measures based on the subjects’ experience during the GBDI were collected. Specifically, the absence of adverse events and the high rates of adherence to treatment, along with the positive answers registered with the CSQ-8, indicate an overall acceptability of the proposed intervention. PwMS with low EDSS and the absence of moderate or severe motor impairment usually do not initiate motor therapy programs, possibly due to a biased perception of their disease. In this sense, high attendance and satisfaction levels suggest a strong interest in this type of intervention. Furthermore, positive feedback was also collected from the semi-structured interview. In general, the participants described the GBDI as enjoyable and stimulating and they highlighted the importance of sharing positive experiences within the group. This sense of connection and support could play a key role in sustaining motivation and active participation throughout the intervention. The main strength of our work lies in the enhancement of the interpersonal sphere by fostering social connection and promoting well-being in pwMS, suggesting that GBDIs could represent a novel, motivating rehabilitation approach with the potential to yield promising outcomes [36].

This study has several limitations that must be acknowledged. The very small sample size (n = 4) strongly restricts the generalizability of the findings and increases the likelihood that results may reflect individual variability rather than the real effects of the intervention. The absence of a control group prevents causal inferences and makes it difficult to distinguish intervention-specific benefits from natural fluctuations in MS symptoms; hence, future randomized studies must account for this facet. Furthermore, the short duration of the intervention does not allow for conclusions to be drawn about the long-term sustainability of improvements, while heterogeneity in disease duration, disability level, and baseline capacities may have influenced responsiveness to training. Eventually, the 66.7% retention rate observed in this pilot underscores the need to implement strategies to maximize retention in future randomized controlled trials, such as jointly agreeing convenient days and times with participants and maintaining regular contact to anticipate and manage potential barriers to attendance. Despite these limitations, the study presents notable strengths. First of all, the strict exclusion criteria, specifically the absence of concurrent participation in other structured rehabilitation programs and no engagement in additional rehabilitative activities by the participants during the intervention period, may have created a relatively controlled context in which clinical improvements could reasonably be attributed, at least in part, to the proposed dance-based program. In addition, to our knowledge, this is one of the first investigations to examine the feasibility of a structured, group-based ballet program in pwMS. The multidimensional assessment approach, encompassing motor, cognitive, and psychosocial domains, enabled a comprehensive evaluation of the outcomes and underscored the potential of dance-based rehabilitation as a multimodal intervention. Furthermore, the fact that all participants completed the program without adverse events supports the safety and acceptability of this approach. These preliminary findings therefore provide a valuable foundation for future randomized controlled trials with larger samples and longer follow-up, aimed at confirming the benefits and refining the clinical implementation of dance-based rehabilitation in MS [35,53]. This study provided preliminary evidence supporting the feasibility, safety, and multifaceted benefits of a group-based ballet intervention in people with multiple sclerosis. By simultaneously targeting motor, cognitive, and psychosocial domains, ballet emerges as a promising and multifactorial rehabilitation strategy that extends beyond conventional physiotherapy [54]. The structured integration of rhythmic movement, choreographed sequences, and social interaction addresses physical impairments while fostering cognitive engagement and emotional well-being.

## 5. Conclusions

The intervention, delivered in a group-based setting, not only promoted motor learning but also facilitated social interaction, peer support, and motivation, factors that are often underrepresented in conventional physiotherapy programs. In this sense, we want to stress the multimodal nature of a GBDI, in which the repeated engagement of overlapping motor and cognitive networks, the aerobic load, and the socially enriching setting may trigger neuroplasticity mechanisms in pwMS. Thus, by simultaneously engaging motor, cognitive, and psychosocial domains, ballet extends beyond conventional physiotherapy, offering a structured yet enjoyable intervention that may enhance functional independence, cognitive resilience, and quality of life. While further research with larger samples and controlled designs is needed to confirm these preliminary results, this study highlights dance-based rehabilitation as a promising adjunct to existing MS care, paving the way for more creative and multidimensional rehabilitation strategies.

## Figures and Tables

**Table 1:** Table illustrating intervention.

Stages	Description	Time
Warm-up	Mobility exercises while standing	10′
Ballet stage and choreography	Balance exercises while standing	30′
	Upper limb exercises while sitting	10′
Final stage	Stretching while sitting	5′
	Stretching while standing	5′

**Table 2:** Clinical and demographic characteristics of the study sample (n = 4).

	Mean (M)	Standard Deviation (SD)	Minimum	Maximum
Age	39.25	6.55	31	45
Gender (male)	2 (2)			
Education	13	4.08	8	13
Disease duration	8.75	1.5	1	10
EDSS	4	0.5	1.5	4.5

**Table 3:** Client Satisfaction Questionnaire (CSQ-8) responses (n = 4).

	Not at All	Slightly	Moderately	Excellent
How would you rate the quality of service you received?	0%	0%	50%	50%
Did you get the kind of service you wanted?	0%	0%	100%	0%
To what extent has our service met your needs?	0%	0%	100%	0%
If a friend were in need of similar help, would you recommend our service to him or her?	0%	0%	50%	50%
How satisfied are you with the amount of help you received?	0%	0%	50%	50%
Have the services you received helped you to deal more effectively with your problems?	0%	25%	75%	0%
In an overall, general sense, how satisfied are you with the service you received?	0%	0%	50%	50%
If you were to seek help again, would you come back to our service?	0%	0%	75%	25%

Values are % of respondents selecting each option.

**Table 4:** Motor outcomes at baseline (T0) and post-intervention (T1).

	T0 Median (Q1–Q3 Quartile)	T1 Median (Q1–Q3 Quartile)
MINI BES-TEST, score	20.00 (16.75–23.25)	23.50 (20.50–26.50)
FFST, seconds	20.56 (13.08–30.51)	11.39 (6.67–14.75)
6MWT, meters	273.50 (232.5–313.5)	293.50 (136.75–348.80)
F8WT, seconds	10.10 (8.20–13.18)	10.00 (3.25–11.50)
BBT (dominant hand), score	29.00 (25.50–33.00)	31.00 (5.00–34.75)
BBT (non-dominant hand), score	30.50 (27.00–34.25)	30.00 (9.25–35.50)
9HPT (dominant hand), seconds	16.94 (14.27–24.22)	18.00 (6.43–21.43)
9HPT (non-dominant hand), seconds	25.15 (21.43–33.48)	35.46 (40.75–62.29)

Legend: FFST = four square step test, 6MWT = 6-min walk-test, F8WT = figure-of-8 walk test, BBT = box and block test, 9HPT = 9-hole peg test.

**Table 5:** Neuropsychological outcomes at baseline (T0) and post-intervention (T1).

	T0 Median (Q1–Q3 Quartile)	T1 Median (Q1–Q3 Quartile)
RAVLT Immediate Recall, score	46.46 (44.46–47.46)	49.17 (37.23–59.95)
RAVLT Delayed Recall, score	9.01 (8.68–9.54)	10.84 (9.54–11.51)
SDMT, score	32.72 (30.33–39.99)	48.22 (39.33–51.49)
TMT-A, time	33.07 (26.56–39.61)	26.07 (21.81–40.11)
TMT-B, time	99.70 (72.49–124.90)	75.16 (60.24–107.1)
MFIS, score	47.00 (39.75–54.75)	40.50 (39.25–41.25)
SF-36 (General Health), score	35.00 (20.00–48.75)	35.00 (28.75–41.25)
SF-36 (Mental Health), score	40.00 (35.00–49.00)	52.00 (50.00–54.00)
SF-36 (Vitality), score	25.00 (23.75–35.00)	40.00 (37.50–43.75)

Legend: RAVLT = Rey auditory verbal learning test, SDMT = symbol digit modalities test, TMT-A = trail making test A, TMT-B = trail making test B, MFIS = modified fatigue impact scale SF-36 = short form health survey.

## Data Availability

The original contributions presented in this study are included in the article. For further inquiries, please contact the corresponding author.

## Appendix A. Individual Demographic and Clinical Characteristics

**Table:**

	Age	Gender	Education	Disease Duration	EDSS
**Subject 1**	31	F	8	7	3.5
**Subject 2**	44	M	13	10	4.5
**Subject 3**	37	F	13	8	3.5
**Subject 4**	44	M	18	10	4.5
**Drop out 1**	35	F	13	10	1.5
**Drop out 2**	45	F	8	1	4
